# Fully Automated Lab-On-A-Disc Platform for Loop-Mediated Isothermal Amplification Using Micro-Carbon-Activated Cell Lysis

**DOI:** 10.3390/s20174746

**Published:** 2020-08-22

**Authors:** Moo-Jung Seo, Jae-Chern Yoo

**Affiliations:** College of Information and Communication Engineering, Sungkyunkwan University, Suwon 440-746, Korea; mtothej92@skku.edu

**Keywords:** lab-on-a-disc, DNA amplification, cell lysis, micro-carbon, infrared camera

## Abstract

Fast and fully automated deoxyribonucleic acid (DNA) amplification methods are of interest in the research on lab-on-a-disc (LOD) platforms because of their full compatibility with the spin-column mechanism using centrifugal force. However, the standard procedures followed in DNA amplification require accurate noncontact temperature control as well as cell lysis at a low temperature to prevent damage to the LOD platform. This requirement makes it challenging to achieve full automation of DNA amplification on an LOD. In this paper, a fully automated LOD capable of performing cell lysis and amplification on a single compact disc of DNA samples is proposed. The proposed system uses micro-carbon to heat DNA samples without damaging the LOD as well as a noncontact heating system and an infrared camera sensor to remotely measure the real temperature of the amplification chamber. Compared with conventional DNA amplification systems, the proposed system has the advantage of full automation of the LOD platform. Experimental results demonstrated that the proposed system offers a stable heating method for DNA amplification and cell lysis.

## 1. Introduction

Over the past few decades, the miniaturization and automation of microfluidics-based analysis protocols have accelerated the development of integrated and low-cost point-of-care (POC) devices for rapid diagnosis. The most notable advantages of using microfluidic devices are the reduced sample consumption, short response time, and high efficiency [1,2]. The performance of microfluidics in POC applications is based on the integration of the key operations on a monolithic device. However, simple independent microfluidic devices cannot perform the overall bioassay protocol processes, thus necessitating an integrated microfluidic POC system.

Currently, numerous microfluidic POC systems are being investigated. As an extension to these systems, a chip design using centrifugal microfluidic technology, which is also known as a lab-on-a-disc (LOD) platform, is proposed herein. LOD platforms are attracting considerable attention in microfluidics engineering [3,4,5,6,7]. A typical centrifugal microfluidic technology controls a series of microfluidic operations, such as aliquoting, valving, sample transport, and mixing, by setting the rotational speed and direction [8,9,10,11,12]. Accordingly, centrifugal microfluidic devices control the fluid by generating the required force for fluid propulsion using a single motor. Thus, an external pump and multiple laboratory instruments are no longer required. Another major advantage of these systems is that the centrifugal force alone regulates the fluid control; therefore, the overall process can be fully automated. Owing to these advantages, temperature control and heating systems can be easily applied to LOD platforms. A heat-sink device is often used as the basis for analytical protocol implementation. Compared with existing centrifugal microfluidics, we expect to achieve full temperature system automation and liquid flow control by applying the proposed design.

Considerable research has been conducted on the polymerase chain reaction (PCR) and loop-mediated isothermal amplification (LAMP) for deoxyribonucleic acid (DNA) amplification. The PCR is the most universal and sensitive DNA amplification method in molecular biology, and in most cases, it amplifies DNA fragments via thermal cycling. However, the PCR is highly error-prone because it comprises complex processes with numerous manual steps. For simplicity, several attempts have been made recently to automatically perform PCR amplification on a single disc for various LOD devices [13,14]. Although these studies reproduced the thermal cycling process of the PCR and yielded good results with regard to DNA amplification, the thermal stability was inadequate [15]. Furthermore, owing to the complex structure of a single disc resulting from the requirement of the actual reproduction of the thermal cycling process, the preprocessing for DNA amplification could not proceed inside the disc. 

LAMP is a single-tube technology for DNA amplification that allows isothermal amplification. Typically, the target DNA sequence for amplification is reacted at a constant temperature of 60–65 °C using three sets of primers and polymerase. This LAMP response is characterized by a significantly larger amount of DNA than that in PCR-based amplification, owing to the specific nature of the activities of the three sets of primers. In contrast to the PCR thermal cycling process, in LAMP, the initial denaturation and three thermal cycles (94, 55, and 69 °C) are not required.

Preprocessing for DNA amplification involves cell lysis. Using a Simplex Easy DNA Kit (Gen-IAL GmbH, Troisdorf, Germany), the entire process (including cell lysis) can be performed in 15 min. The processing time was shortened to approximately one-fourth of that when the cell lysis is performed via the conventional method [16,17,18,19,20]. However, it has a drawback: the temperature must be increased to 95 °C. This is a high temperature for an LOD system and may damage the proposed disc, which may cause loss of the DNA sample. Thus, the valve system is required to be robust.

Heating systems have been studied to perform point-of-need DNA detection inside LOD devices. Abkar Ahmed Sayad et al. [21], proposed an LOD device for specifically detecting *Salmonella*. They achieved the temperature for the LAMP reaction by using a hot air gun and measured the temperature of the LOD surface using an infrared (IR) thermometer. The experimental setup consisted of a temp-plate sensor capable of calibrating three temperatures: 60, 65, and 82 °C. However, in this system, the hot air gun increased the temperature not in a specific local area but over a wide range, and it increased the ambient temperature to 250 °C; thus, the safety of the peripheral devices needs to be ensured. Des Brennan et al. [22], proposed a noncontact IR heating system with fluorescence detection for detecting *Haemophilus influenzae* ribonucleic acid (RNA) samples using nucleic acid sequence-based amplification. The noncontact heating system can control the temperature between 20 and 70 °C. However, to control the IR heater, a design and an additional device are needed for maintaining a constant distance between the disc and the heater; the system cannot control the temperature above 70 °C. Jacky Fong-Chuen Loo et al. [23] developed an LOD platform that required a low-operational power heating system for detecting genetically modified papayas using LAMP. The disc was attached to the thermistor, and the temperature of the disc was controlled. However, this only increased the temperature of the surface; thus, additional energy was needed to increase the temperature in the chamber. Additionally, although this method is adequate for maintaining the temperature in the chamber at 65 °C, it is not suitable for increasing the temperature to >90 °C.

We solved these problems by using micro-carbon (CEP21KS, PCT Co., Gumi, Korea). Micro-carbon has the advantage of local heating; thus, the LOD platform is not damaged. Moreover, micro-carbon reduces the difficulty in valve design because the local heating reduces the vapor pressure. Accordingly, with the use of micro-carbon, the entire lysis chamber does not need to be heated. A laser is used for heating [24], and because the micro-carbon is pure black, it efficiently absorbs most of the heat energy (IR light) generated by the laser diode. These features lead to rapid heating of the lysis chamber. Thus, this paper presents an LOD platform on which a sample solution undergoes a fully automated protocol once it is manually injected into the disc.

## 2. Materials and Methods

### 2.1. Processing System

**Figure 1:**
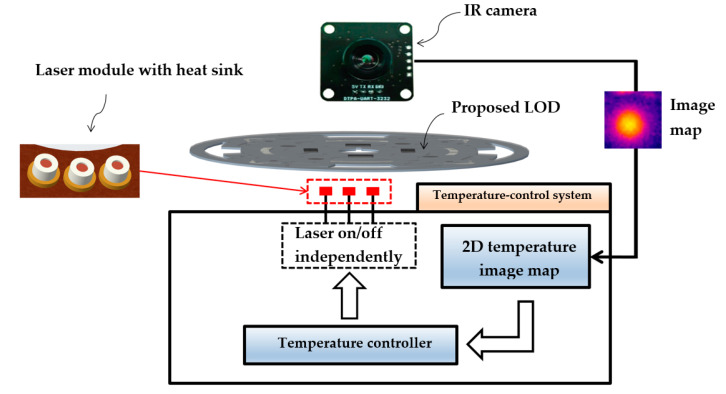
Temperature-control system for the amplification chamber on the lab-on-a-disc (LOD). The infrared (IR) camera provides a two-dimensional (2D) temperature map showing the distribution of the temperature in the amplification chamber. The laser module is turned on or off to keep the temperature stable.

The proposed design of the LOD system is presented in Figure 1. The system is equipped with one laser module and one IR camera sensor. The laser module, consisting of three laser diodes (QL80T4HD-Y, QSI, Cheonan, Korea), is the key device for valve control, and conducts cell lysis at 95 °C and LAMP heating at 60 °C. The laser module uses computer-based pulse-width modulation control with a duty cycle, and each laser diode can be turned on/off independently. The laser module is not fixed; it can be moved to a desired position, thereby enabling the application of the operations of the entire valve control and heating chambers to the LOD. Using an IR camera (DTPA-UART-3232, Diwell Electronics Co., Ltd., Gunpo, Korea), the temperature of the amplification chamber during the LAMP amplification process is measured in real time, and the chamber temperature are controlled to maintain the target temperature during the amplification; this is achieved using a two-dimensional (2D) temperature image map and the laser module on/off control. The image map is transferred to a computer and analyzed using a MATLAB program (MathWorks, Natick, MA, USA), and the temperature of the chamber is determined every 0.5 s.

### 2.2. Disc Fabrication

**Figure 2:**
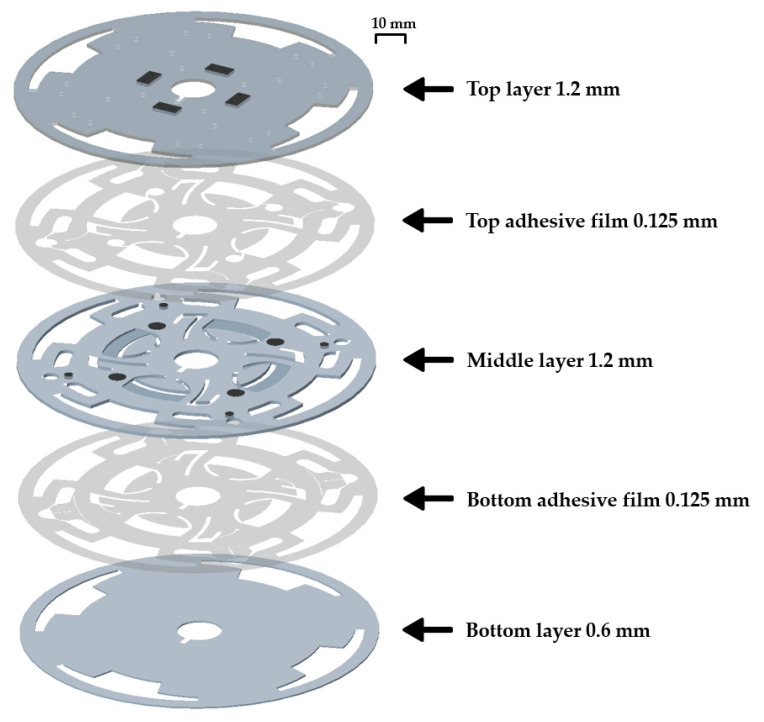
LOD layer assembly schematic. The top and middle layers are bonded with the top adhesive film; the middle and bottom layers are bonded with the bottom adhesive film.

The proposed LOD platform was fabricated with five stacked layers, which were composed of three polycarbonate layers and two double-sided adhesive films (Figure 2). All the layers had outer and inner diameters of 120 and 15 mm, respectively, for the driving motor (Swiss Amiet, Seoul, Korea). The three polycarbonate layers, which were carved using a computer numerical control machine (PromillEZ200, Protek Co., Daejeon, Korea), were bonded using the two double-sided adhesive films (Tesa 4928, Tesa, Norderstedt, Germany; thickness: 0.125 mm), which were designed using a laser-cutting machine (Gravograph, Rillieux-la-Pape, France). The top and middle layers were each 1.2 mm thick, and the bottom layer was 0.6 mm thick.

**Figure 3:**
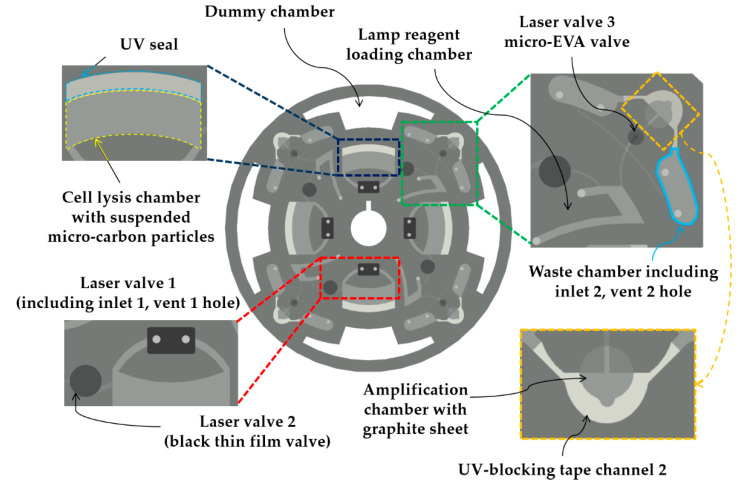
Disc fabrication top-view assembly using the Creo 4.0 Computer-Aided Design program. The disc was fabricated via axial patterning at a 90° angle to allow DNA detection four times on one disc. Differences from other designs are indicated by a dotted line or rectangle, and the name of each component is shown. Insets present detailed explanations where necessary.

A detailed design of the disc was drawn using the Creo 4.0 (PTC Korea, Seoul, Korea) software program (Figure 3). All five layers had a dummy chamber designed to eliminate unnecessary space in the disc design. To reduce the effects of the experimental procedure on the disc, the dummy chamber was designed with a 5 mm safety distance from the surrounding structures. The main purpose of the dummy chamber was to reduce the total disc weight and noise during the rotation of the disc.

**Figure 4:**
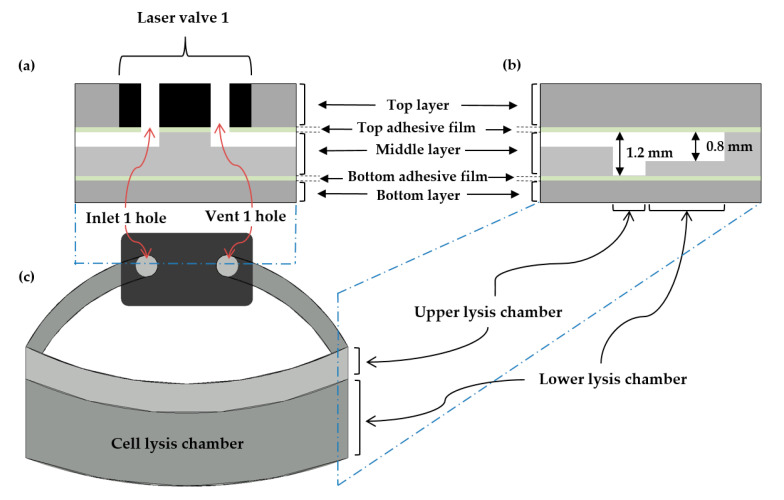
Schematic of laser valve 1 and the cell-lysis chamber. (**a**) Front view of laser valve 1, showing that the inlet and vent have an all-in-one form; thus, they can be simultaneously closed with one shot of the laser beam. (**b**) Right-side view of the cell-lysis chamber, showing the different heights of the internal structure, which consists of the upper (1.2 mm thick) and lower (0.8 mm thick) lysis chambers. This height differential allows the capillary-induced force to block the liquid from flowing out toward the channel. (**c**) Top view of laser valve 1 and the cell-lysis chamber.

In the proposed LOD design, laser valves 1–3 absorb the laser beam with high efficiency, leading to easy melting. Laser valve 1 is made of ethylene vinyl acetate (EVA), as in our previous study [24]. However, in the present study, in contrast to the previously designed structure, the inlet and vent holes are combined (Figure 4). Laser valve 2 is the opening valve and is also called the laser burst valve [25]. It blocks the pathway, but is easily opened when the laser beam is irradiated to hold the sample reagent in the cell-lysis chamber until the lysis proceeds. Laser valve 3, which is made of EVA (similar to the closing valve) and called the micro-EVA valve [26], was originally the channel of the cell-lysis chamber supernatant and LAMP preparation reagent to the amplification chamber. Before the LAMP heating was started (60 °C), laser valve 3 was closed using the laser module.

Inlets 1 and 2 are used to inject liquid into the disc; each inlet is designed with a vent hole to maintain the internal pressure when the liquid is injected. Inlet 1 is in the cell-lysis chamber and can be used for loading ultraviolet (UV) adhesives (UV-3300, Skycares, Gimpo, Korea) into the outer boundary of the cell-lysis chamber, which was called the UV seal, or for loading reagents needed for cell lysis. Inlet 2 is in the waste chamber and can be used for loading UV adhesives into the outer boundary of the DNA amplification chamber or for storing excess liquid. Inlet 2 is placed in the same shape on the right and left sides of the amplification chamber to inject UV adhesives into the UV blocking tape channel in both directions via the capillary effect. Both UV adhesives enforce the sealing strength of the following chambers: The cell lysis and DNA amplification chambers [26,27].

The cell-lysis chamber (volume = 200 µL) was sufficiently large to accommodate a typical DNA sample with a volume of 100 µL. The LAMP preparation reagent chamber and the amplification chamber had volumes of 50 and 25 µL, respectively. The volume of the waste chamber, which included the inlet 2 and vent 2 holes, was 300 µL on each side.

### 2.3. Preparation of Samples and Reagents

In this study, *Salmonella typhimurium* (IB 5379 Tanzania, original ID 73-37671) was used as the target pathogen sample to demonstrate the performance of the proposed LOD system. The *Salmonella typhimurium* (OD600 value = 1.0) sample was cultured in 25 mL of tryptic soy broth and incubated overnight at 37 °C [24]. Each incubated *Salmonella* sample was aliquoted into a tube with a volume of 1 mL. Each sample was aliquoted into 1 mL cultured *Salmonella* samples. Before being used in the LOD system, the unnecessary supernatants of the aliquots were removed via washing twice using phosphate-buffered saline (PBS) and centrifugation at 13,000 rpm for 1 min. Subsequently, a Simplex Easy DNA kit was used for preparations for the experiments on the LOD system.

### 2.4. LAMP Reagent Reaction

Similar to a previous study conducted by our research group [27], the LAMP reaction compound had a total volume of 25 µL and comprised 1.6 µM of forward inner primer (FIP)/backward inner primer (BIP) primers, 0.2 µM of F3/B3 primers, and 0.8 µM of loop forward (LF)/loop backward (LB) primers, as well as the following components: 10× Isothermal Amplification Buffer II (B0374S, New England Biolabs, MA, USA), 8U of Bst 3.0 DNA Polymerase (M0374S, New England Biolabs, MA, USA), 6 mM MgSO_4_, and 1.4 mM dNTP Mix (Deoxynucleoside Triphosphate Set, Roche, Mannheim, Germany). With reference to Cho et al. [28], the primer set was prepared using the following sequences, targeting the invA gene:

5′-ATGATGCCGGCAATGGCGTCAGCCAGCTTTACGGTTCCT-30(FIP),
5′-GATGACCCGCCATGGTATGGATCCATCACCGATGGTCAGC-30(BIP),
5′-GAAGCGTACTGGAAAGGGAA-30(F3),
5′-GGGATCTGGGCGACAAGA-30(B3),
5′-TTGATAAACTTCATCGCACCGT-30(LF), and
5′-TTATCCTCCGCGCTGTCTACTTA-30(LB).

### 2.5. DNA Lysis Experimental Procedure for Disc

Table 1 presents the stepwise sequence of the DNA cell lysis performed using the LOD platform before LAMP heating. For applying the centrifugal force, a motor was used to generate the required number of revolutions per min. The loading of the sample was the main part of step 1; however, there was a possibility that the DNA would remain in the channel leading to the cell-lysis chamber. To minimize this liquid leakage, the LOD performed step 2, in which a rotation was conducted at 5000 rpm for 30 s, as specified in the table. In step 2, all of the DNA remaining in the channel was transferred to the cell-lysis chamber. Laser valves 1, 2, and 3 and the cell-lysis laser module, as shown in Figure 1, were in operation in each process. Step 4, which required a temperature of 95 °C for 10 min, was performed using micro-carbon and a laser module for cell lysis. After the cell lysis, the DNA and carbon were separated via centrifugation for 5 min (step 5). The entire sequence of processes was completed in approximately 18 min, including 15 min for the cell lysis.

### 2.6. Micro-Carbon Used as Incubator

**Figure 5:**
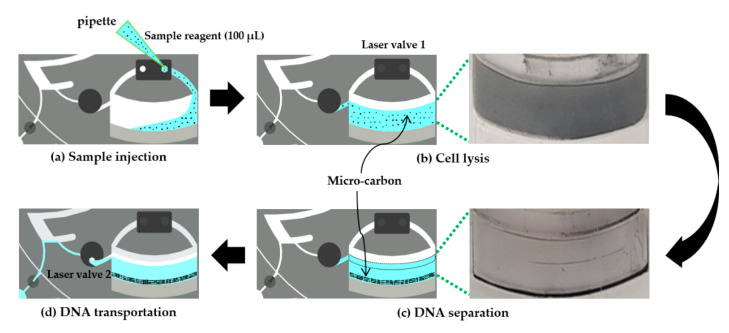
Micro-carbon-activated cell lysis for DNA extraction from the sample solution. (**a**) Sample injection: the sample solution containing micro-carbon is injected through the inlet hole. (**b**) Cell lysis: laser valve 1 is closed, and subsequently, the cell-lysis chamber is heated for 10 min. (**c**) DNA separation: DNA is centrifugally separated from the debris and micro-carbon at 11,000 rpm for 5 min. (**d**) DNA transportation: laser valve 2 is opened and, subsequently, the supernatant (approximately 5 µL of DNA) flows to the next chamber (the amplification chamber) as the LOD rotates.

Micro-carbon was used to increase the temperature in the lysis chamber. The prepared DNA sample solution (total volume of 100 µL) was homogeneously mixed with 0.2 g of micro-carbon, which was composed of particles having an average size of 18 µm. Through an inlet channel, the mixture was then injected into the lysis chamber, where the sample solution was heated. A vent channel was designed to maintain constant pressure in the lysis chamber during the injection of the sample solution (Figure 5).

Because the Simplex reagent was used, the chamber was heated at 95 °C for 10 min during the cell lysis. Generally, when the temperature of a liquid increases to 95 °C, which is close to the boiling point of water, the kinetic energy of the molecules in the liquid state increases, increasing the internal pressure. Although the inlet and vent holes made of EVA were designed to prevent leakage, there was a risk that the blocking function would be lost above 80 °C [24]. Thus, increasing the temperature to 95 °C led to the leakage of sample reagent from the lysis chamber. 

**Figure 6:**
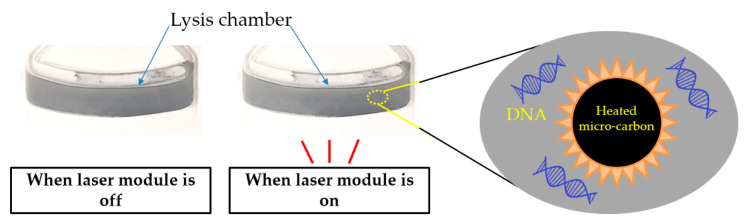
Micro-carbon-activated cell lysis. The micro-carbon absorbs a laser beam with high efficiency; subsequently, the DNA molecules are released from the heated cells when the laser module is on.

To address this problem, pure black micro-carbon was used in the heating system. By not transferring the heat at 95 °C—a temperature that EVA cannot withstand—to the outside and instead retaining the heat, the micro-carbon increased the heat of only the liquid inside the lysis chamber, which was needed to reach the target temperature (Figure 6). To verify the performance of micro carbon, a Heat stick (Thermomelt Heat-Stik 5 in 203 °F/95 °C, Markal, Elk Grove Village, IL, USA) and an incubator (OF-11, JeioTech, Daejeon, Korea) were used.

### 2.7. LAMP Using Graphite Sheet and IR Camera

**Figure 7:**
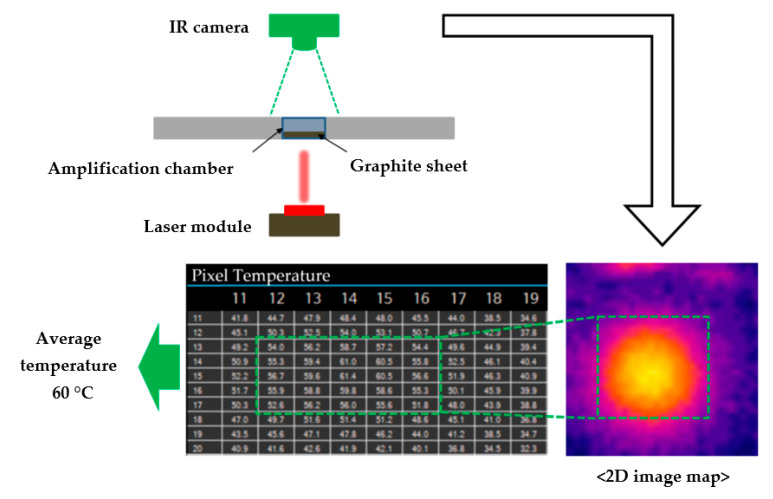
Two-dimensional image map to measure the average temperature of the amplification chamber. The image map was obtained from the IR camera installed above the LOD, and the graphite sheet inside the chamber efficiently absorbed the laser beam. The laser module was turned on or off to maintain the average temperature of the chamber at 60 °C.

In addition to implementing cell lysis using micro-carbon-activated heating for fully automated DNA detection on the LOD, DNA amplification was a main objective of the present study. The Loopamp DNA Amplification Kit (Eiken Chemical Co., Ltd., Tokyo, Japan) and a graphite sheet (graphite depth: 0.1 mm, GENetech, Seoul, Korea) were used because the LAMP process was performed at 60 °C. Figure 7 shows the location of the graphite sheet on the disc. To illustrate its function, a side-view schematic of the graphite is also presented.

Graphite is a substance with a high heat of fusion, melting, and solidification at a certain temperature; thus, it can store and release large amounts of energy. When graphite is transformed into sheet form (hereinafter referred to as a “G-sheet”) with a thickness of a few micrometers, its state becomes very sensitive to temperature changes. For a G-sheet, the phase change occurs at a temperature of 60 °C. In this study, the IR camera measured the temperature distribution as a 2D image map.

## 3. Results and Discussion

### 3.1. Micro-Carbon Performance

**Figure 8:**
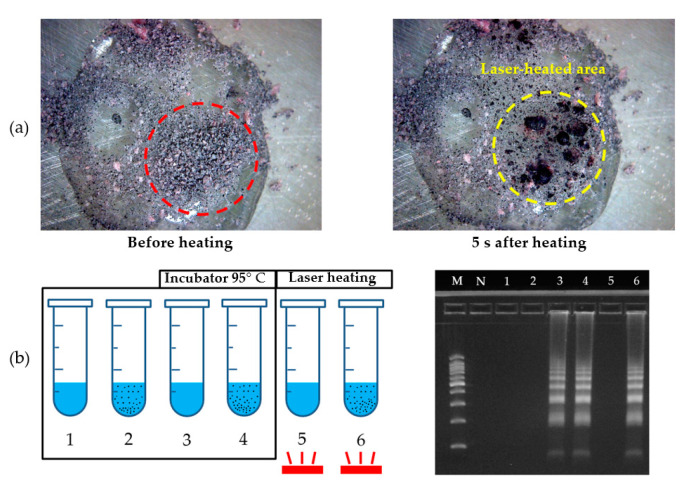
Stability and efficiency test of micro-carbon with DNA. (**a**) Before and after water laser heating with the heat-stick reacting at 95 °C and micro-carbon. The reaction occurred in 5 s, and the heat-stick reaction was used to prove that the micro-carbon reached 95 °C when the laser beam was on. (**b**) All the numbered tubes underwent the same experimental procedure, except for the lysis heating method. Tubes 1 and 2 contained only the sample solution (no DNA), whereas tubes 3–6 contained DNA and sample solutions. Micro-carbon was added to tubes 2, 4, and 6. Tubes 1–4 were heated using an incubator set to 95 °C. A laser beam was applied to tubes 5 and 6. The bottom-right figure shows the electrophoresis result for each tube.

This study was designed such that cell lysis could be performed without leakage inside the LOD using micro-carbon. The leakage problem was efficiently addressed by the internal structural design of the disc. However, to achieve the intended results, it was necessary to confirm whether the micro-carbon, which played a key role in the cell lysis, reached 95 °C when the laser was emitted. We used a heat stick to determine the temperature, which was indicated by a color change. The sample solution used in cell lysis was a mixture of 100 µL of a DNA sample solution (initial concentration: 10^8^ CFU/mL), 0.2 g of micro-carbon, and 0.2 g of the heat stick ground into powder. When the heat-stick powder reached 95 °C, it turned from pink to dark pink (Figure 8a). Local heating occurred only in the portion where the laser was targeted.

Furthermore, as we designed an experimental protocol in which micro-carbon was directly mixed with a sample solution, it was also necessary to verify the biochemical stability of the micro-carbon. The proposed disc was not used for this; rather, we used the spin-column method with a tube, which is a conventional DNA detection method. To determine whether the micro-carbon affected the sample solution and DNA, different test sample solutions were prepared, with and without DNA. Additionally, to determine whether laser heating had a direct effect on the micro-carbon, cell lysis was performed using an incubator and laser heating. Subsequently, LAMP amplification and electrophoresis were performed. The results are shown in Figure 8. The electrophoresis results indicated the biochemical stability of the micro-carbon. The local heating system using a laser module with micro-carbon exhibited a similar performance as the incubator.

### 3.2. G-Sheet and IR Camera

**Figure 9:**
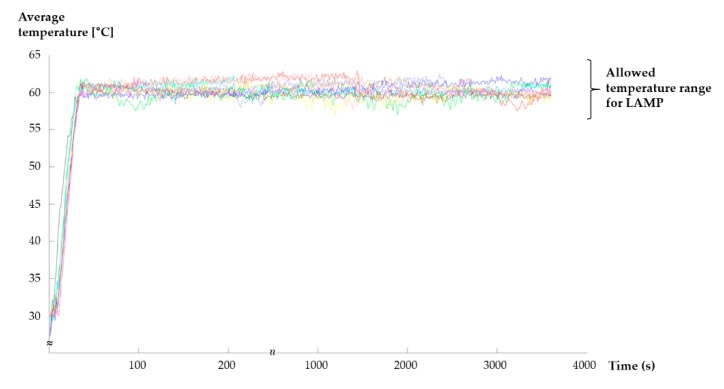
Graph showing that the amplification chamber reached approximately 60 °C in <1 min (resolution: 0.1; maximum value: 62.9; minimum value: 57.0).

The LAMP reaction requires heating at 60 °C for 60 min. We used a G-sheet to perform this heating on the LOD and a camera to ensure that this temperature was maintained for 60 min. Using the temperature image map from the camera, the real-time temperature was determined throughout the 60 min duration of the LAMP reaction. Then, using the temperature-control system shown in Figure 1, the temperature in the amplification chamber was maintained at 60 °C. In total, 10 runs were performed to ensure the reproducibility of the results, which are presented in Figure 9. The results indicated that the time required for the LAMP chamber to reach a temperature of 60 °C was only 32 s (standard deviation of 2.1705). Furthermore, Figure 9 shows that the temperature in the LAMP chamber was maintained between 57 and 63 °C, which is the temperature range required for a stable reaction [15,29].

### 3.3. Verification of LAMP Results

**Figure 10:**
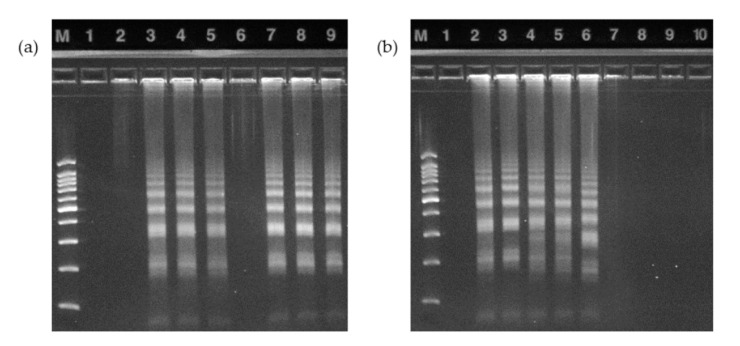
Electrophoresis results. M: DNA size marker (SizerTM-100bp, INtRON Bio., Seongnam-si, Korea); 1st lane: Negative (no DNA template in sample tube). (**a**) Electrophoresis photograph of 10-fold dilution; 2–10th lanes: LAMP product with an initial concentration of 10^8^ CFU/mL; 10-fold dilution occurs with increasing location number. (**b**) Photograph comparing the electrophoresis results of the proposed LOD and the existing spin column; 2nd lane: LOD reference (sample solution without target DNA); 3–5th lanes: LAMP reaction using the LOD; 6th lane: Spin-column reference (sample solution without target DNA; same as 1st lane); 7–9th lanes: LAMP reaction using the spin column.

The *Salmonella typhimurium* DNA template was used, and the corresponding target primer was set. To verify the aforementioned LAMP reaction, we observed the results of electrophoresis analysis after the LAMP reaction of the target DNA using a spin-column method with a tube. The 10-fold dilution results shown in Figure 10a prove that the LAMP response presented in this paper is adequate for detecting foodborne pathogens, including *Salmonella*.

To verify the LAMP amplification using the automated LOD, an electrophoresis analysis was performed, and the results were compared with the spin-column results obtained using a tube (Figure 10b). With the intention of removing the experimental variables to the greatest extent possible, as shown in Figure 3, a total of four electrophoresis results were obtained using one LOD. In one case of electrophoresis, a sample solution without the addition of target DNA was used to serve as a reference for the disc. Similarly, for the electrophoresis using a spin column, a sample solution without target DNA was employed as a reference. The analysis results proved that LAMP can be performed in the LOD using micro-carbon. Additionally, a hydroxynaphthol blue dye can be added to the LAMP reaction, which allows colorimetric analysis, for confirming the presence or absence of amplification inside the LOD platform [27].

## 4. Conclusions

An LOD study was conducted using a new heating system that satisfies the platform automation and miniaturization requirements for molecular diagnostic devices, which is an essential goal of microfluidic POC systems. Using the newly designed LOD, cell lysis, and DNA amplification can be performed automatically. An electrophoresis analysis was performed, and the results were compared with those of a spin-column analysis to verify the results.

In previous studies [24], we implemented complete cell lysis and DNA amplification inside the disc, but the two processes were conducted independently. In the present study, full automation of the molecular diagnostic device was achieved through complete integration of the cell-lysis and DNA-amplification processes.

The novel micro-carbon design used in the present study allowed local heating inside the disc. Thus, even if the temperature of the sample inside the disc reached 95 °C, the necessary process could proceed without damage or leakage. The main advantage of the heating system used in the proposed method is that it is easy to obtain and apply. If the characteristics of micro-carbon are utilized well, it is expected to play a very important role not only in the LOD field but also in various other fields involving microfluidic flows.

## Figures and Tables

**Table 1:** Summary of the LOD-platform experimental procedure for DNA cell lysis.

Step	Procedure	Revolutions per Minute (RPM)	Time (s)
1	Load the sample with micro-carbon into the cell-lysis chamber.	−	−
2	Spin the LOD to move the mixture remaining in the channel leading to the cell-lysis chamber.	5000	30
3	Laser valve 1 melting—close the inlet and vent concurrently.	−	30
4	Incubate for 10 min at 95 °C by heating micro-carbon using the cell-lysis laser module (warm-up time: 30 s).	−	630
5	Centrifuge the LOD for 5 min to separate the DNA.	11000	300
6	Laser valve 2 melting—open the channel leading to the amplification chamber.	−	10
7	Load the loop-mediated isothermal amplification (LAMP) preparation reagent and transfer it to the amplification chamber with the supernatant of the cell-lysis chamber.	10000	30
8	Laser valve 3 melting—close the channel leading to the LAMP heating.	−	10
